# Determination of the Oxygen Content in the LiF–NaF–KF Melt

**DOI:** 10.3390/ma16114197

**Published:** 2023-06-05

**Authors:** Anna A. Maslennikova, Peter N. Mushnikov, Alexey V. Dub, Olga Yu. Tkacheva, Yury P. Zaikov, Ya-Lan Liu, Wei-Qun Shi

**Affiliations:** 1 Institute of High Temperature Electrochemistry, Ural Branch of the Russian Academy of Sciences, Academicheskaya Str., 20, 620990 Ekaterinburg, Russia; 2 JSC “Science and Innovations”, State Corporation Rosatom, 32/2 Kadashevskaya Quay, 115035 Moscow, Russia; 3 Institute of High Energy Physics, Chinese Academy of Sciences, No. 19B Yuquan Road, Shijingshan District, Beijing 100049, China

**Keywords:** LiF–NaF–KF, melt, voltammetry, method of reduction melting

## Abstract

The present paper is dedicated to the quantitative determination of oxygen-containing impurities in the LiF–NaF–KF eutectic using electrochemical (cyclic and square-wave voltammetry) and reduction melting methods. The LiF–NaF–KF melt was analyzed before and after purifying electrolysis. The amount of oxygen-containing impurities removed from the salt during purification was determined. It was found that after electrolysis, the concentration of oxygen-containing impurities decreased by 7 times. The results obtained via electrochemical techniques and reduction melting were well-correlated, which made it possible to evaluate the quality of the LiF–NaF–KF F melt. To verify the analysis conditions, mechanical mixtures of LiF–NaF–KF containing Li_2_O were analyzed using the reduction melting method. The oxygen concentration in the mixtures varied from 0.672 to 2.554 wt. %. Based on the analysis results, the dependence approximated by the straight line was obtained. These data may be used to draw calibration curves and to further develop the procedure of oxygen analysis of fluoride melts.

## 1. Introduction

Currently, the technology and design of molten-salt nuclear reactors (MSRs) with a fuel salt based on fluoride melts are under active development. The MSR is one of the six concepts of “Generation IV” nuclear reactors. Lizin et al. [1] presented an overview of the nuclear reactor concepts, in which molten salts are supposed to be used. MSRs have a number of advantages [2,3,4,5]: energy efficiency due to higher temperatures; the possibility of fuel salt composition variation during the reactor’s operation, including fuel reprocessing and the introduction of transuranium elements for efficient transmutation; and the inherent safety of reactors of this type. MSRs are often considered as reactors with enhanced (natural) safety features. One of the reasons for this is that the fuel in MSRs is in a liquid state; thus, it is easy to ensure natural safety against the overheating of the reactor. In emergency scenarios, the solid plug in the reactor is melted, and the fuel is drained into a containment trap. The second reason is the constant removal of gaseous fission products and the constant replenishment of fresh fuel. This makes it possible to avoid delivering fuel with a large reactivity margin into the reactor and hence reduces the risks of uncontrolled reactor runaway. The third reason is the low pressures in the reactor vessel, which provides increased safety.

Molten alkali and alkali earth fluorides are commonly used as components of fuel mixtures. They must meet the following requirements [2]:

- A low liquidus temperature to ensure an operating temperature of 500–750 °C;
- A low neutron capture cross-section, as the melt must be resistant to reactor radiation;
- Low corrosivity, as the melt must be relatively inert with respect to the construction materials;
- Low viscosity, as hydrodynamic properties become more important when working with coolants in a secondary cycle;
- High heat capacity and electrical conductivity, because the melt is required to have thermophysical and hydrodynamic properties that ensure its operation as a heat exchange fluid without excessive power consumption for circulation;
- Low vapor pressure over the entire range of operating parameters.

The following salt mixtures are those most often studied as promising fuel salts or coolants for MSRs: LiF–BeF_2_, LiF–NaF–BeF_2_, LiF–NaF–KF, NaF–KF, NaF–NaBF_4_, LiCl–KCl–MgCl_2_, and NaNO_3_–KNO_3_ [1,6,7,8]. Among the other salt systems that have been proposed, the eutectic melt of lithium, sodium, and potassium fluorides (0.465LiF–0.115NaF–0.420KF, i.e., FLiNaK) has several advantages in terms of the physicochemical properties, as well as an increased ability to dissolve actinide and lanthanide fluorides [5,9,10,11]. At the same time, special requirements are imposed regarding the quality of prepared fluoride melts to be used as a working medium in MSRs [1,12,13]. Particular attention is paid to oxygen-containing impurities due to their significant effect on corrosion processes [14,15,16,17,18].

There are two approaches to the quantitative analysis of oxygen in fluoride melts: in situ methods and methods that require melt sampling. Among the in situ methods, the electrochemical methods are considered promising for both oxygen content analysis and its removal [19,20,21]. The disadvantage of such methods is the difficulty of the results’ interpretation, especially if the system under study contains several impurity elements with similar electrochemical characteristics. Nevertheless, the electrochemical procedures using sensors for the determination of the content of elements in molten electrolytes are rather widely represented [19,21,22,23]. Wang et al. [24] used voltammetry to determine the oxygen content (in concentration limits of 200–1000 ppm) in FLINaK containing different amounts of Li_2_O. Voltammograms were obtained on spectrally pure graphite with a potential sweep rate of 0.05 V/s. It was found that the dependence of the peak current on the oxygen ion concentration was linear. However, it was not possible to plot the dependence of the peak current on the Li_2_O concentration in the LiF–NaF–KF melt considering the current–voltage (CV) results obtained for the FLiNaK-Li_2_O melt in [23]. The reasons for this difference may be the use of different electrochemical devices and analysis techniques, as well as different materials of the working electrode. For example, Manning et al. [25] only managed to obtain voltammograms with a clear anodic peak for LiF–NaF–KF and LiF–BeF_2_ melts with a gold working electrode. On the other hand, the possible influence of the cathode process on the CV dependencies was noted in [22].

Square-wave voltammetry is a type of CV analysis [26] in which the polarization of the working electrode is carried out by shifting its potential with “rectangular” overvoltage pulses. As a response of the system, the currents of the direct and reverse processes, as well as the resulting current, are recorded. In comparison with classical chronovoltammetry, square-wave voltammetry makes it possible to reduce the effects of changes in the concentrations of oxidized and reduced electroactive ions participating in the electrode process. In practice, this manifests in the amplification of the recorded signal of the resulting current and the formation of more distinct current peaks. It should be noted that using square-wave voltammetry, Shen et al. [19] managed to determine a low concentration of oxygen ions in LiF–NaF–KF equaling 49 ppm. 

Among the methods that require sampling, reduction melting in a carrier gas atmosphere is the most interesting, since it provides express results and has a developed hardware base [27,28]. The principle of reduction melting is that the oxygen-containing sample is placed in a graphite crucible and heated rapidly in an inert gas flow to a high temperature; the oxygen ions are reduced by carbon to CO/CO_2_ and, in the carrier gas flow, are transferred to an IR detector for the quantitative determination of oxygen. The fundamentals of the method were developed mainly to determine the oxygen concentration in ceramics and metals [29,30,31].

There is a limited number of publications devoted to the use of the reduction melting method for the determination of oxygen in molten salts. The principle of this method does not change; however, features such as the volatility of salts at high temperatures and thermal decomposition before the reduction come to the fore. 

To study the solubility of oxides and oxychlorides (MgO, Nd_2_O_3_, NdOCl) in chloride and chloride–fluoride melts, the authors of [21,27,32] developed a technique for the carbothermal reduction of oxygen in salts using a LECO instrument. This technique was used by Shen et al. [19] to analyze commercial FLiNaK for the oxygen content. The total concentration of oxygen impurities was found to be 679 ppm. After blowing a HF/H_2_ gas mixture through molten FLiNaK at 500 °C for 48 h, the oxygen concentration decreased to 83 ppm.

However, the analysis of fluoride systems, such as the LiF–NaF–KF eutectic, is accompanied by a number of difficulties associated with the relatively low melting points of salts. In addition, standard samples with the corresponding fluoride matrix have not yet been developed [31].

Since there is currently no certified method for controlling the oxygen concentration in fluoride melts, the purpose of this work is to consider various methodological approaches aiming to solve the problem of oxygen analysis for molten salts based on the LiF–NaF–KF eutectic mixture. Specifically, we aim to evaluate the method of reduction melting in an inert gas flow and the electrochemical in situ methods of cyclic and square-wave voltammetry.

## 2. Materials and Methods

Reagents Preparation

To prepare a mixture of LiF–NaF–KF with different ratios of individual salts, 46.5, 11.5, and 42 mol. %, respectively, the following reagents were used:

- Commercial chemically pure lithium fluoride powder provided by JSC VECTON, St. Petersburg, Russia. The mass fraction of LiF is no less than 99.5%.
- Commercial extra-pure sodium fluoride powder provided by LAVERNA Ltd., Moscow, Russia. The mass fraction of NaF is no less than 99.0%.
- Commercial extra-pure acid potassium fluoride powder provided by JSC REACHEM, Moscow, Russia. The mass fraction of KF·HF is no less than 99%.

In accordance with the chemical certificates provided by the above suppliers, the total concentration of oxygen-containing impurities (in the form of SO_4_^2−^) in these salts was ~0.1 wt. %, and the total concentration of the main cationic impurities (Mg, Ca, Si, Al, Fe, Pb) was ~0.25 wt. %.

The preparation procedure of the LiF–NaF–KF eutectic mixture, in which an anhydrous acid KF·HF salt is used instead of KF, has been described in detail elsewhere [9,10]. The principle of this method is to melt the initial reagents in a glassy carbon crucible in air through stepwise heating up to 750 °C, while KF·HF decomposes, forming anhydrous potassium fluoride (KF) and HF vapor. The HF vapors interact with impurities in the initial salts; this interaction results in the purification of the melt. The obtained cooled melt was repeatedly heated to 300–350 °C, and then it was transported to the glove box with an inert argon atmosphere (the contents of water and moisture did not exceed 1 ppm).

**Figure 1:**
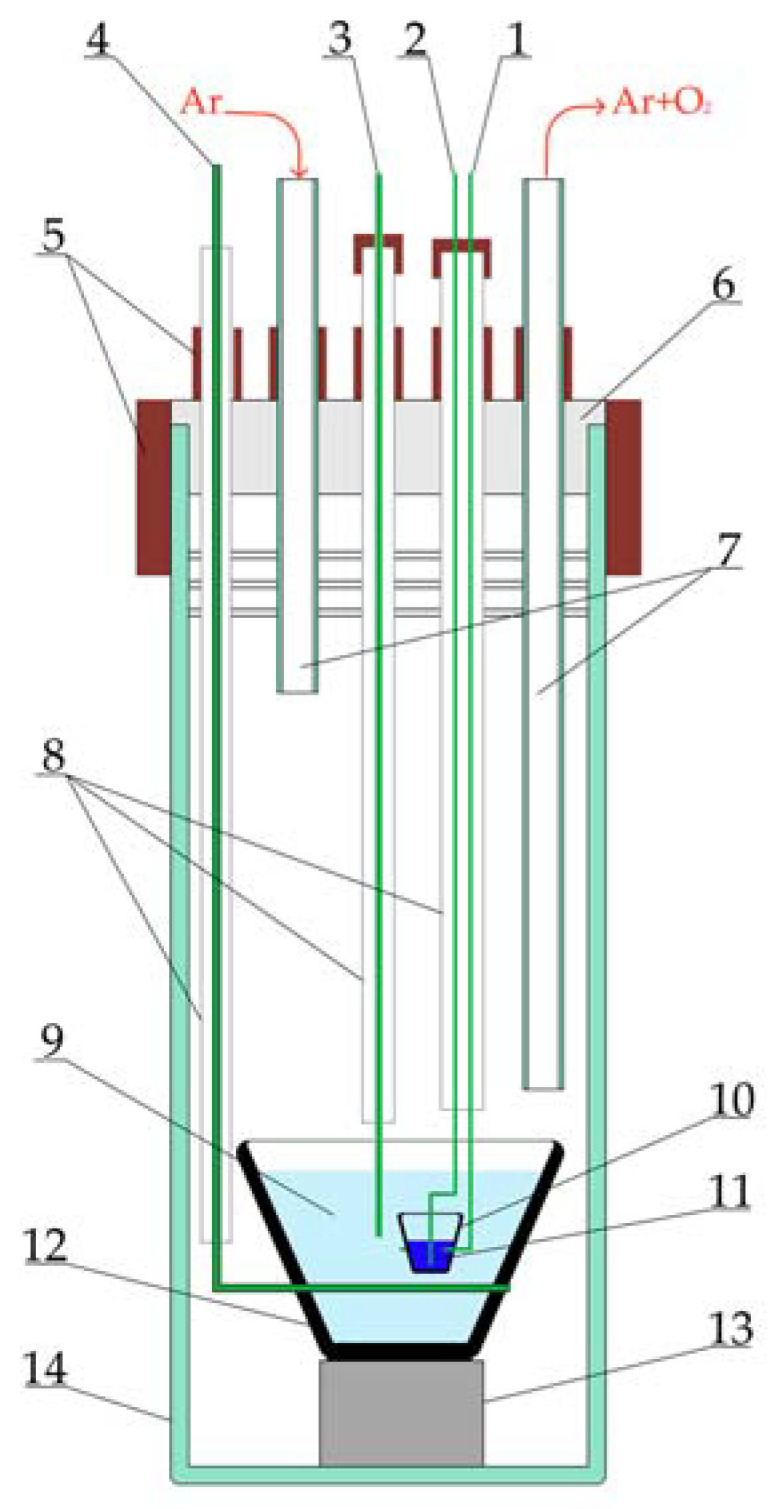
Schematic of the purification electrolysis setup: 1—molybdenum cathode suspension; 2—molybdenum cathode current lead; 3—molybdenum wire (quasi-reference electrode); 4—molybdenum anode current lead; 5—vacuum rubber seals; 6—fluoroplastic cover; 7—gas outlets; 8—alundum straws; 9—studied melt; 10—graphite crucible; 11—molten bismuth; 12—glassy carbon crucible; 13—boron nitride stand; 14—stainless steel retort.

The obtained LiF–NaF–KF eutectic was purified via purifying electrolysis. This process ensures the effective purification of the melt of oxygen ions [24]. The experimental setup for purifying electrolysis is presented in Figure 1. This setup was composed of a stainless-steel retort with a sealed fluoroplastic cover. A glassy carbon crucible containing the melt was applied as an anode. Oxygen ions were oxidized at the anode with the formation of CO/CO_2_ and were removed with the argon flow. A graphite crucible with molten bismuth, immersed in the melt, served as a cathode. During electrolysis, metallic potassium was deposited on the cathode. A molybdenum wire of 1 mm in diameter was used as a quasi-reference electrode. A glassy carbon crucible with the LiF–NaF–KF melt was placed in the retort, which was connected to a gas vacuum system and located in a resistance furnace. The interior of the retort was evacuated to a residual pressure of 5 Pa and filled with high-purity argon. The cell area was continuously blown using an argon flow with a rate of 1 L/min. 

Purifying electrolysis was carried out at 650 °C in a galvanostatic mode, gradually reducing the current from 50 to 20 mA. The current was reduced when a sharp increase in the anode potential caused by the anode effect was recorded.

The cooled retort with the salt was placed into a glove box with an inert argon atmosphere after electrolysis. All further procedures and manipulations were carried out in the glove box to avoid contact of the melt with air. 

**Figure 2:**
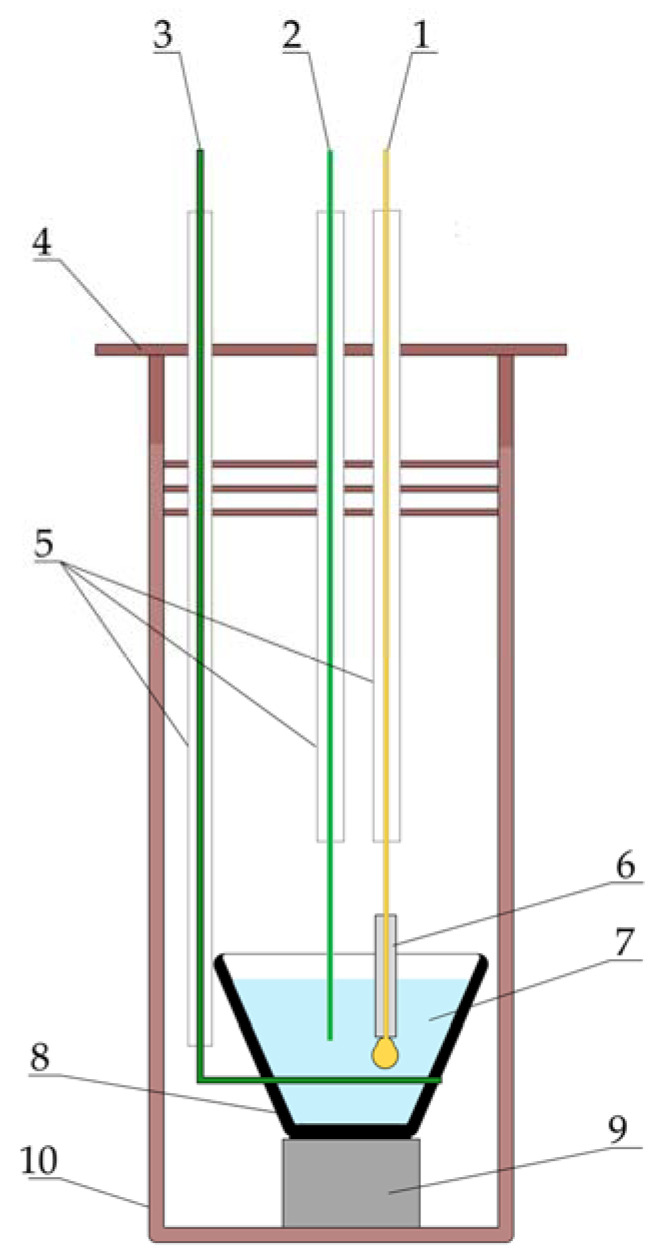
Schematic of the electrochemical cell: 1—drop-like gold electrode (anode); 2—molybdenum wire (quasi-reference electrode); 3—molybdenum current lead; 4—nickel screens; 5—alundum straws; 6—boron nitride straws; 7—studied melt; 8—glassy carbon crucible; 9—boron nitride stand; 10—stainless steel retort.

The content of oxygen impurities in the obtained melt was estimated via cyclic and square-wave voltammetry using a three-electrode electrochemical cell, which consisted of a drop-shaped gold working electrode with a surface area of ~65 mm^2^. Figure 2 is an illustration of this electrochemical cell. The lower part of the working electrode was insulated using a boron nitride straw to eliminate the influence of processes occurring at the three-phase electrode–melt–argon interface. A glassy carbon crucible with a melt served as an auxiliary electrode, and a molybdenum wire was used as a quasi-reference electrode. All measurements were carried out at 650 °C using an Autolab PGSTAT 302 N potentiostat/galvanostat with Nova 1.12 software.

The LiF–NaF–KF melt samples were analyzed via the method of reduction melting using an oxygen analyzer, METAVAK-K (NPO EXAN, Russia), that included a measuring unit and an extraction furnace. The essence of the reduction melting method is the interaction between the carbon of the crucible and oxygen of the analyzed sample at high temperatures. Such interaction results in the formation of CO gas that is blown by the gas flow into the extraction furnace filled with CuO reagent, where it transforms into its analytically active form, CO_2_. The obtained gas is analyzed using a non-dispersion IR detector. The optic density, which is proportional to the concentration of the oxygen based on the detector, is determined using the analytical signal. After the measurements, the IR detector provides an evolution pattern with the peak, which the square area is the analytic signal. Based on this signal, having determined the resulting characteristics and weight of the sample, the mass fraction of oxygen is calculated with the device software.

To determine the oxygen concentration, the following analysis conditions were experimentally selected: the temperature of 1590 °C and helium used as a carrier gas. The sample was placed in a tin capsule. The instrument was calibrated using a LECO Powder Iron LCRM standard with the oxygen content ω(O) = 1.10 ± 0.02%.

The FLiNaK samples were prepared in a glovebox in an inert gas atmosphere at oxygen and moisture concentrations not exceeding 1 ppm. The salt ingots were ground into powder. This powder was sampled into tin capsules, with approximately 50 mg of powder in one sample. After that, the capsule containing the powder was thoroughly sealed using two tweezers via stepwise folding and pressing.

## 3. Results

### 3.1. Purifying Electrolysis

**Figure 3:**
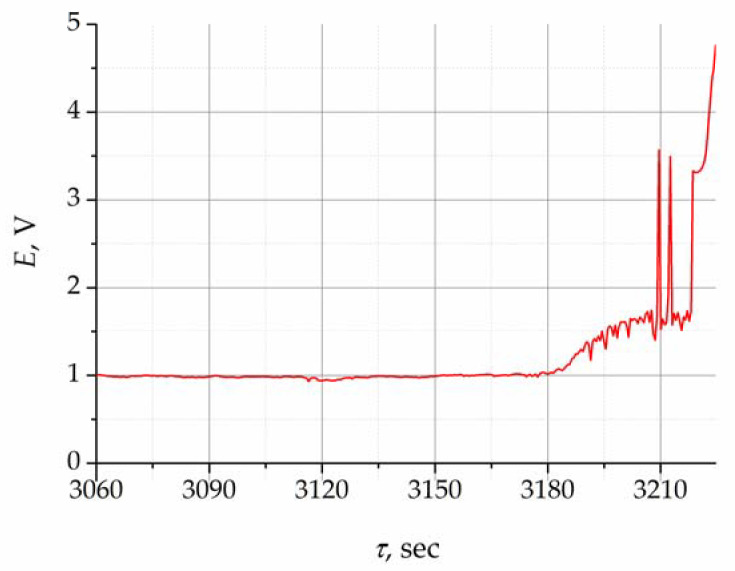
Change in potential during purifying electrolysis.

A change in potential during purifying electrolysis performed at 30 mA (the calculated anode current density is around 2 mA/cm^2^) is presented in Figure 3. During electrolysis, the gas bubbles form, grow, and detach from the surface of the working electrode, which leads to constant change in the anode area and, accordingly, in the anode current density. This is indicated by slight potential oscillations around the value of 1 V.

The concentration of oxygen ions in the melt decreased to the limit value, and alternative reactions associated with the formation of fluorine and carbon compounds occurred. This was accompanied by the increase in potential starting from 3180 s, as illustrated in Figure 3. Furthermore, the electrode potential shifted sharply to the positive side, exceeding 2 V, which was associated with the process of fluorine ion oxidation in the melt and the appearance of the anode effect. This potential jump signifies the end of the purifying electrolysis process at a given current.

Upon the completion of electrolysis, the interior of the retort was purged with argon for 5 h in order to remove all the gaseous products. Then, the furnace was cooled to room temperature, the retort was dismantled in the glove box, and the solidified bead of the melt was removed.

Assuming that the anode current efficiency was 100%, the mass of released oxygen at the anode was calculated according to the Faraday law:

(1) $$m=\frac{Q\cdot M}{F\cdot n},$$

where *m* is the mass of released oxygen at the anode, *Q* is the amount of electricity passed through the melt during electrolysis, and *M* is the molar mass of oxygen.

Using the calculated mass of released oxygen, we calculated the decrease in the concentration of the oxygen-containing impurities during purification electrolysis, which amounted to 0.05 wt. %.

### 3.2. Cyclic and Square-Wave Voltammetry

**Figure 4:**
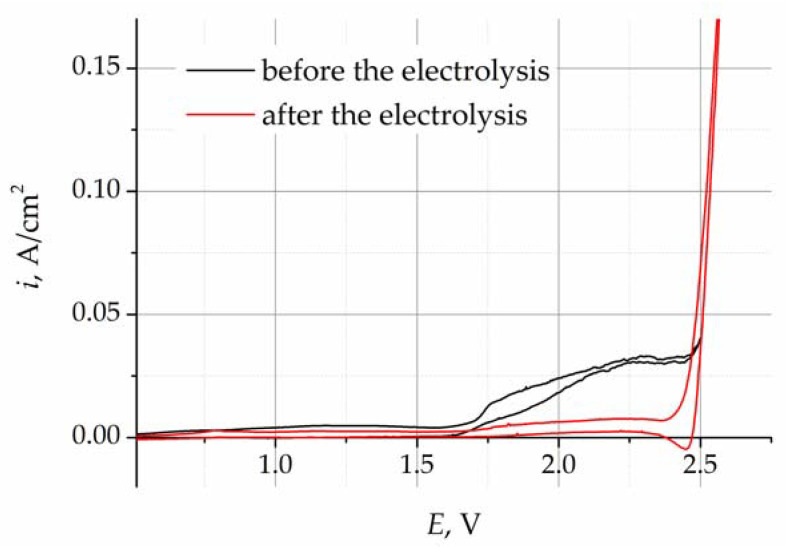
Cyclic voltammograms of the LiF–NaF–KF melt before and after purification electrolysis.

The cyclic voltammograms (CVs) obtained for the LiF–NaF–KF melt before and after purification electrolysis are presented in Figure 4. The CVs were recorded at a potential sweep rate of 100 mV/s. In the voltammograms obtained for the melt before purification electrolysis, the increase in current density at potentials more positive than 1.6 V corresponds to the process of oxygen ion oxidation in the gold working electrode. The current density steps observed in the CVs indicate the formation of gaseous oxygen in the working electrode, according to reaction (2). After the potential of 2.4 V, the current density increases sharply, which is associated with the dissolution of the gold electrode, according to reaction (3).

(2) $$O^{2-}-2e^{-}=\frac{1}{2}O_{2\;(g)};$$

(3) Au^0^ − e^−^ = Au^+^.

The CVs of the melt after the purification electrolysis illustrate that the current density, corresponding to the release of oxygen, decreases to the background value, which designates a significant decrease in the concentration of oxygen impurities.

Cyclic voltammetry makes it possible to qualitatively assess the presence of oxygen-containing impurities in the melt; however, this method is not appropriate for quantitative analysis, since it is difficult to determine the current density of the oxygen oxidation process. The CV curves do not have any clearly pronounced peaks that could be used to define the current density of the peak corresponding to the oxidation of oxygen ions. One of the methods of voltammetry analysis allows one to decrease the impact of the concentration alterations in the oxidized and reduced ions that take part in the studied electron process. In practice, this is illustrated by more pronounced signals and the formation of clearer peaks. 

**Figure 5:**
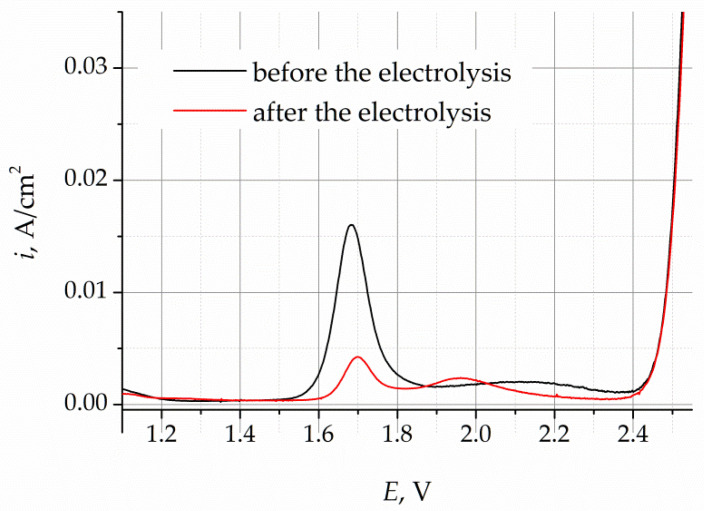
Square-wave voltammograms of the LiF–NaF–KF melt before and after purifying electrolysis.

The square-wave voltammograms (SWV) obtained for the LiF–NaF–KF melt before and after purification electrolysis, which were recorded at a sweep frequency of 100 Hz, are presented in Figure 5. A distinct peak in the potential range of 1.5–1.9 V and a wave at potentials more positive than 2.4 V can be observed. The latter refers to the process of the working electrode’s dissolution, according to reaction (1), while the peak refers to the process of oxygen oxidation, which is in good agreement with the reported literature data [16]. The current density of the peak corresponding to the oxygen evolution in the melt after purification electrolysis decreased from 16 to 4 mA/cm^2^.

**Figure 6:**
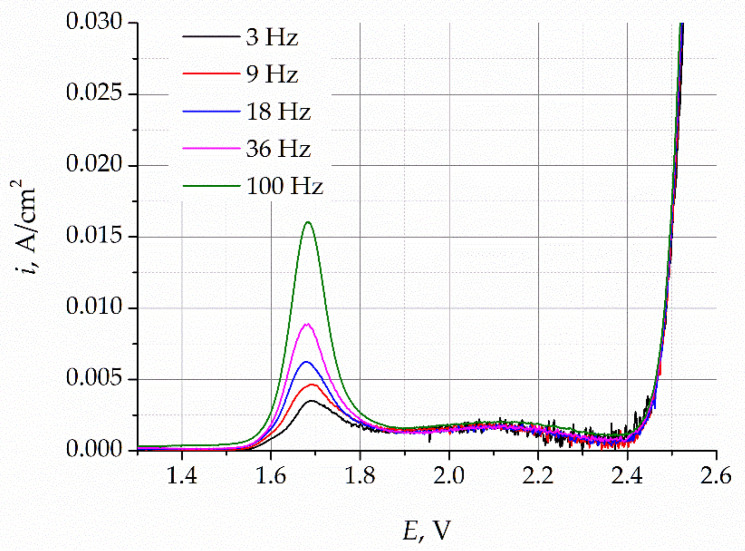
SWVs obtained for the LiF–NaF–KF melt before purification electrolysis of the gold electrode at different sweep rates.

To establish the nature of the oxidation process, the SWVs were recorded at different sweep frequencies, as presented in Figure 6.

**Figure 7:**
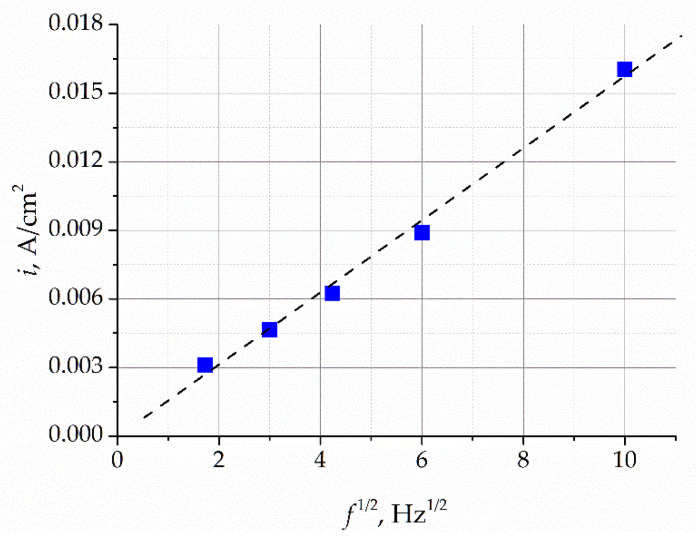
Dependence of the peak current on the square root of the sweep frequency.

The increase in the potential sweep frequency results in an increase in the peak current density, while the peak potential remains unchanged. The dependence of the intensity of the current peaks on the square root of the sweep frequency is shown in Figure 7. This dependence is approximated by a straight line, which indicates that the process of oxygen ion oxidation in the gold electrode is limited by the process of oxide ion diffusion in the electrode. It follows from these data that the limiting current of the peaks is directly proportional to the concentration of oxygen ions and can thus be used to analytically estimate the content of oxygen impurities in the melt.

The number of electrons involved in the oxidation process was calculated using the following equation [26]:

(4) $$W_{\frac{1}{2}}=3.52\cdot \frac{R\cdot T}{n\cdot F},$$

where $W_{\frac{1}{2}}$ is the peak halfwidth, *R* is the universal gas constant, *T* is the temperature, *n* is the number of electrons that take part in the reaction, and *F* is the Faraday constant.

The calculated value of the number of electrons participating in the oxidation process is 2.15, which corresponds to reaction (2). Thus, it can be argued that the SWV peak corresponds to the oxygen oxidation reaction at the anode, and the peak current density can be used to estimate the quantitative content of oxygen ions in the LiF–NaF–KF melt.

### 3.3. Reduction Melting

The results of the oxygen analysis of the LiF–NaF–KF melt samples taken before and after purifying electrolysis, which were obtained through the reduction melting method using an oxygen analyzer METAVAK-K, are presented in Table 1.

According to the results given in Table 1, the oxygen concentration in the LiF–NaF–KF samples before purification was 0.0461 wt%, and after purification it decreased by 7 times and amounted to 0.0068 wt%. The data obtained are consistent with those calculated according to the Faraday law.

To confirm the applied analysis technique, (LiF–NaF–KF)-Li_2_O mixtures were prepared. LiF–NaF–KF salt containing 0.0068 wt% of oxygen after purification electrolysis was mixed with different amounts of Li_2_O. To ensure the homogeneity of the samples under study, the LiF–NaF–KF powders were mixed with Li_2_O powder in a Retsch-400 vibration mill for 5 h. The compositions of samples with different oxygen concentrations are presented in Table 2.

The calculated values of the oxygen content presented in Table 2 consider 0.0068 wt. % ppm of oxygen in the purified LiF–NaF–KF and 53.43 wt. % of oxygen in the lithium oxide.

Based on the fault level calculations for each of the mixture samples, it can be concluded that the discrepancy between the theoretical and experimental (average) values of the mass fraction of oxygen-containing impurities in the samples ranges from 0.0068 wt. % to 2.544 wt. % and does not exceed 1%. The error of determination is the maximum for the n^0^1 and n^0^4 mixture samples, with theoretical contents of oxygen impurities of 0.0068 wt. % and 1.942 wt. %, respectively. The minimum error of determination was obtained for the n^0^3 mixture sample and amounted to 2.976%.

**Figure 8:**
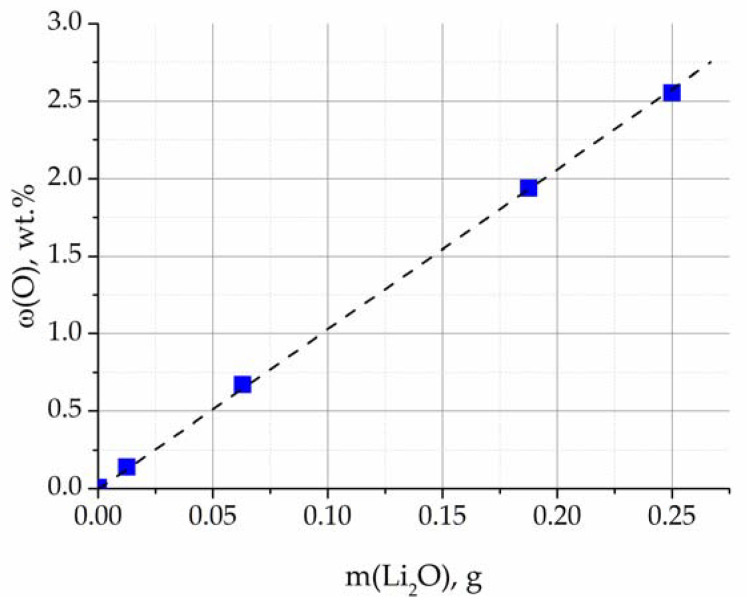
Ratio of the Li_2_O mass and oxygen concentration in the LiF–NaF–KF-Li_2_O mixture.

The ratio of the Li_2_O mass and the oxygen concentration in the studied mixtures, obtained via reduction melting, is shown in Figure 8. The dependence is approximated by a straight line, which verifies the selected analysis conditions.

## 4. Conclusions

The melting of the original components LiF, NaF, and KF·HF in air makes it possible to obtain a LiF–NaF–KF molten eutectic with an oxygen-containing impurity concentration of 0.05 wt. %. Further purification of the LiF–NaF–KF melt via electrolysis in a galvanostatic mode using a glassy carbon crucible as an anode is an effective way to purify fluoride salt melts of oxygen-containing impurities and reduce their concentration by 6–7 times.

The content of oxygen-containing impurities in the LiF–NaF–KF eutectic was determined before and after purifying electrolysis using the methods of square-wave and cyclic voltammetry, as well as reduction melting. The measurements were carried out using a drop-shaped gold working electrode. The obtained CV curves illustrated the wave, which allowed for a qualitative determination of the oxygen-containing impurities in the melt. The obtained square-wave voltammetry curves demonstrated a clear peak, the intensity of which was proportional to the oxygen content in the melt. Thus, in combination with the method of reductive melting, the concentration of oxygen-containing impurities in the LiF–NaF–KF eutectic melt after purification electrolysis was estimated and amounted to 0.0068 wt. %.

The content of oxygen impurities in the melt before electrolysis was determined according to the amount of electricity consumed for electrolysis via calculations following the Faraday law, as well as the method of reduction melting. According to the results obtained using both methods, this value was approximately 0.05 wt. %.

Since there are currently no certified methods for the determination of oxygen-containing impurities in the salt systems of LiF–NaF–KF compositions based on the method of reduction melting, it was necessary to ensure that the analysis was performed correctly under the selected conditions. Therefore, mechanical LiF–NaF–KF mixtures with known Li_2_O concentrations were prepared and analyzed. The results obtained correlate well with the theoretical values.

The considered approaches to the analysis of the oxygen content in LiF–NaF–KF salt, i.e., electrochemical analysis and reduction melting, are promising and can be used to determine even low oxygen concentrations in fluoride salts.

## Figures and Tables

**Table 1:** Oxygen content in the LiF–NaF–KF samples before and after purifying electrolysis.

№	Before Purification		After Purification	
	Sample Mass, mg	Oxygen Concentration, wt%	Sample Mass, mg	Oxygen Concentration, wt%
1	48.4	0.0425	39.2	0.0066
2	39.5	0.0363	41.5	0.0071
3	37.8	0.0464	63.9	0.0063
4	43.8	0.0442	48.5	0.0076
5	43.5	0.0609	57.4	0.0062
Average concentration, wt%		**0.0461 ± 0.01**	**0.0068 ± 0.0007**	
Standard deviation, wt%		0.009	0.0006	

**Table 2:** Compositions of the (LiF–NaF–KF)-Li_2_O mixtures.

Mixture Number	m_mixture_, g	m_Li2O_, g	ω_theor_(O)_mixture_, %	ω_exp_(O)_mixture_, %
1	5.000	0	0.0068	0.0066
				0.0071
				0.0063
				0.0076
				0.0062
	Standard concentration, wt. %			**0.0068 ± 0.0007**
	Standard deviation, wt. %			**0.0006**
	Relative standard deviation, %			**8.824**
2	5.0125	0.0125	0.141	0.0147
				0.0128
				0.0145
	Standard concentration, wt. %			**0.140 ± 0.028**
	Standard deviation, wt. %			**0.01**
	Relative standard deviation, %			**7.142**
3	5.063	0.063	0.673	0.0658
				0.0663
				0.0696
	Standard concentration, wt. %			**0.672 ± 0.0057**
	Standard deviation, wt. %			**0.002**
	Relative standard deviation, %			**2.976**
4	5.1875	0.1875	1.942	2.131
				1.875
				1.814
	Standard concentration, wt. %			**1.940 ± 0.467**
	Standard deviation, wt. %			**0.169**
	Relative standard deviation, %			**8.711**
5	5.25	0.25	2.556	2.555
				2.430
				2.677
	Standard concentration, wt. %			**2.544 ± 0.342**
	Standard deviation, wt. %			**0.123**
	Relative standard deviation, %			**4.835**

## Data Availability

Data sharing is not applicable to this article.
